# Photocatalytic Oxidation of Acetone Over High Thermally Stable TiO_2_ Nanosheets With Exposed (001) Facets

**DOI:** 10.3389/fchem.2018.00175

**Published:** 2018-05-18

**Authors:** Ting Shi, Youyu Duan, Kangle Lv, Zhao Hu, Qin Li, Mei Li, Xiaofang Li

**Affiliations:** ^1^Key Laboratory of Catalysis and Materials Science of the State Ethnic Affairs Commission & Ministry of Education, Hubei Province, College of Resources and Environmental Science, South-Central University for Nationalities, Wuhan, China; ^2^College of Chemistry and Chemical Engineering, Wuhan University of Science and Technology, Wuhan, China

**Keywords:** TiO_2_, photocatalytic degradation, acetone, fluorine, oxygen vacancy

## Abstract

Anatase TiO_2_ (A-TiO_2_) usually exhibits superior photocatalytic activity than rutile TiO_2_ (R-TiO_2_). However, the phase transformation from A-TiO_2_ to R-TiO_2_ will inevitably happens when the calcination temperature is up to 600°C, which hampers the practical applications of TiO_2_ photocatalysis in hyperthermal situations. In this paper, high energy faceted TiO_2_ nanosheets (TiO_2_-NSs) with super thermal stability was prepared by calcination of TiOF_2_ cubes. With increase in the calcination temperature from 300 to 600°C, TiOF_2_ transforms into TiO_2_ hollow nanoboxes (TiO_2_-HNBs) assembly from TiO_2_-NSs via Ostwald Rippening process. Almost all of the TiO_2_-HNBs are disassembled into discrete TiO_2_-NSs when calcination temperature is higher than 700°C. Phase transformation from A-TiO_2_ to R-TiO_2_ begins at 1000°C. Only when the calcination temperature is higher than 1200°C can all the TiO_2_-NSs transforms into R-TiO_2_. The 500°C-calcined sample (T500) exhibits the highest photoreactivity toward acetone oxidation possibly because of the production of high energy TiO_2_-NSs with exposed high energy (001) facets and the surface adsorbed fluorine. Surface oxygen vacancy, due to the heat-induced removal of surface adsorbed fluoride ions, is responsible for the high thermal stability of TiO_2_-NSs which are prepared by calcination of TiOF_2_ cubes.

## Introduction

Semiconductor photocatalysis has attracted much attention due to its potential applications such as water (Regmi et al., 2018; Xu et al., 2018) and air purification (Wen et al., 2015; Cui et al., 2017; Li et al., 2017a; Qi et al., 2017) and water splitting for clean H_2_ energy (Cheng et al., 2018) due to its peculiar chemical and physical properties. As a typical semiconductor photocatalyst, anatase TiO_2_ (A-TiO_2_) usually shows excellent photocatalytic activity. However, it usually transforms into poor photoreactive rutile TiO_2_ (R-TiO_2_) when calcination temperature is higher than about 600°C, which hampers the practical applications of A-TiO_2_ in hyperthermal situations (Lv et al., 2011; Liang et al., 2017). For example, the temperature of some industrial waste gases after burning can be as high as 900–1000°C, which makes the photocatalytic oxidation technology lose its power due to the phase transformation of A-TiO_2_ at such high temperature. There are many photocatalytically active stable TiO_2_-coated ceramic materials which are used for the control of organic contaminants, including sanitary wares, bathroom tiles and self-cleaning glass. They require high processing temperatures and therefore need excellent stability at high temperature (Periyat et al., 2009). Thus, exploration of thermally stable TiO_2_ photocatalyst is very important but this question remains unsolved.

As one of volatile organic compounds (VOCs), acetone is a widely used solvent especially in chemical plant. The emission of acetone can not only cause the problems to global environment, but also bring harms to human health (Zhu et al., 2016). When the concentration of acetone vapor is higher than 500 ppm, it can irritate eyes and discomfort respiratory system (Li et al., 2018). So, it is of great importance to explore an efficient method to decompose of acetone. Because semiconductor photocatalysis is considered as an environmentally benign way, it is no doubt very promising for VOCs removal under normal conditions.

It is generally accepted that the quantum efficiency of photocatalysis is highly related to the physical properties of TiO_2_ such as crystal structure, relative crystallinity, size of the particle, and specific surface area (Xu et al., 2007; Lan et al., 2015; Wang et al., 2015, 2016; Wen et al., 2015; Li et al., 2016; Sajan et al., 2016; Liang et al., 2017; Xia et al., 2017; Lin et al., 2018a,b). Recently, growing interesting has been devoted to the synthesis of TiO_2_ nanocrystals with exposed high energy {001} facets by surface fluorination of TiO_2_ to reduce the surface energy (Yang et al., 2008, 2009; Chen et al., 2010; Yu et al., 2014; Sajan et al., 2016; Liang et al., 2017). On considering that TiOF_2_ cubes are fluorine-containing materials, they prefer to transform into high energy TiO_2_ nanocrystals during calcination. After the removal of surface adsorbed fluorine ions at high temperature, the formed oxygen vacancy (OV) on the surface of the photocatalyst will prevent the fusion of neighboring A-TiO_2_ nanocrystals, which is believed to improve the thermal stability of TiO_2_ nanocrystals. Herein, we systematically studied the dependence of the structure and photocatalytic activity of TiOF_2_ cubes on the calcination temperature. Acetone was selected as the targeted VOCs to evaluate the photoreactivity of the as-prepared photocatalyst.

## Experimental section

### Synthesis

Precursor TiOF_2_ was synthesized through the solvothermal reaction of Tetrabutyl titanium (TBT), acetic acid (HAc) and hydrofluoric acid (HF) (Huang et al., 2013). Typically, 20.0 g of TBT was dropwise added into the mixed solution containing 6.4 ml of HF (47 wt%) and 40.0 ml of HAc under magnetic stirring. The resulted white suspensions were transferred to an autoclave with volume of 100 ml, which was then kept at 200°C for 2 h. The white deposition was collected and washed with ethanol and distilled water until the filtrate is neutral (pH7). After oven dry at 80°C, we obtained the precursor TiOF_2_.

Precursor TiOF_2_ was then calcined at certain temperature (300-1200°C) for 2 h by keeping the same heating rate (5°C min^−1^). The obtained photocatalyst is denoted as Tx (Table 1), where x is the calcination temperature. For example, T500 is that the photocatalyst which was prepared by calcination of the precursor TiOF_2_ at 500°C for 2 h.

**Table 1:** Physical property of the photocatalyst.

**Sample**	**Calcination temperature (°C)**	**Phase structure*^a^***	**S_BET_*^b^* (m^2^g^−1^)**
T300	300	TiOF_2_	5.7
T400	400	TiOF_2_/A-TiO_2_	6.4
T500	500	TiOF_2_/A-TiO_2_	5.6
T600	600	A-TiO_2_	3.2
T700	700	A-TiO_2_	2.4
T800	800	A-TiO_2_	2.0
T900	900	A-TiO_2_	2.0
T1000	1000	A-TiO_2_/R-TiO_2_	1.3
T1100	1100	A-TiO_2_/R-TiO_2_	1.2
T1200	1200	R-TiO_2_	0.4

a *A-TiO_2_ and R-TiO_2_ represent anatase TiO_2_ and rutile TiO_2_, respectively*.

b *The BET surface area is determined by a multipoint BET method using the adsorption data in the relative pressure (P/P_0_) range from 0.05 to 0.3*.

### Characterization

XRD patterns of the photocatalysts were performed on a X-ray diffractometer (D8- advance, Bruker Co., German), and the scan rate of Cu K*a* radiation keeps 0.02° 2θ s^−1^, using an accelerated voltage and applied current of 15 kV and 20 mA, respectively. We observe the morphology of the prepared photocatalyst by an FESEM with an acceleration voltage of 20 kV (Hitach, Japan) and a TEM (Tecnai G20, USA) using an acceleration voltage of 200 kV, respectively. The optical property of the photocatalyst was measured by a spectrophotometer (UV-2550, Shimadzu, Japan) from 200 to 800 nm using BaSO_4_ as background. FTIR was obtained on a infrared spectrometer (NeXUS 470) using the KBr pellet technique. XPS was recorded using monochromatic Al-Ka radiation under vacuum at 2 × 10^−6^ Pa on a photoelectron spectrometer (VG Multilab 2000). The C1s peak at 284.8 eV originated from the surface adventitious carbon is used to reference all the binding energies. EPR signal of the photocatalyst was recorded in an EPR spectrometer (JES-FA 200, JEOL) at room temperature (frequency of 100 kHz and microwave power of 0.99 mW). Nitrogen sorption isotherm was measured on an ASAP 2020 nitrogen adsorption equipment (Micromeritics, USA). Before investigating the surface areas of the photocatalysts, all samples were degassed firstly at 200°C.

### Photoelectrochemical measurements

We use CHI760e as electrochemical workstation (Shanghai, China) to measure the transient photocurrent, EIS Nyquist plots and Mott-Schottky plots in a standard there-electrode system, where Pt wire was used as the counter electrode, and the prepared samples and Ag/AgCl in saturated KCl were used as the working and reference electrode, respectively. During the measurement, 0.4 M Na_2_SO_4_ was used as electrolyte solution. In the Mott-Schottky measurement, direct current potential polarization was kept at a fixed frequency, and the working electrode was prepared on a glassy carbon electrode. Before the test of photocurrent, 50 mg photocatalysts and 30 μL Nafion were dispersed into 1 mL water/absolute ethanol mixed solvent (v/v = 1/1), and then the mixed aqueous solution was dispersed uniformly through ultrasound to form a homogeneous catalyst colloid. The ITO/TiO_2_ electrode was prepared using the as-prepared photocatalyst colloid as precursor by a drip coating method. The light source is a 3W LED lamp (Shenzhen LAMPLIC, China) emitted mainly at 365 nm. The intensity for the lamp at working distance is measured to be 0.41 W/cm^2^.

### Evaluation of the photocatalytic activity

Photocatalytic oxidation of gasous acetone was used to evaluate the photocatalytic activity of the photocatalyst, which was performed in a 15 L reactor at ambient temperature under UV light irradiation. 0.3 g of the powder was firstly dispersed in 30 mL of double distilled water by sonicating treatment for 5 min. The obtained suspensions were then evenly transferred into three glass dishes in diameters of about 7.0 cm. After drying at 80°C for about 2 h, the dishes that have been coated with a layer of the photocatalyst were placed in the reactor, and then 10 uL of acetone was injected into the reactor by a microsyringe. The vaporated acetone then begins to adsorb on the surface of the photocatalyst. Thirty minutes later, the adsorption–desorption equilibrium of acetone can be established. The photocatalytic oxidation of acetone begins after turning on the UV light, which is 5 cm above the dishes. The concentrations of acetone and the produced carbon dioxide in the reactor were determined online with a Photoacoustic IR Multigas Monitor (Model 1412, INNOVA). Before irradiation, the initial concentration of acetone after the adsorption equilibrium was about 300 ppm, which almost keeps unchanged for about 5 min before lighting a UV lamp (15W@365 nm).

## Results and discussion

### Effect of calcination temperature on phase structure

**Figure 1:**
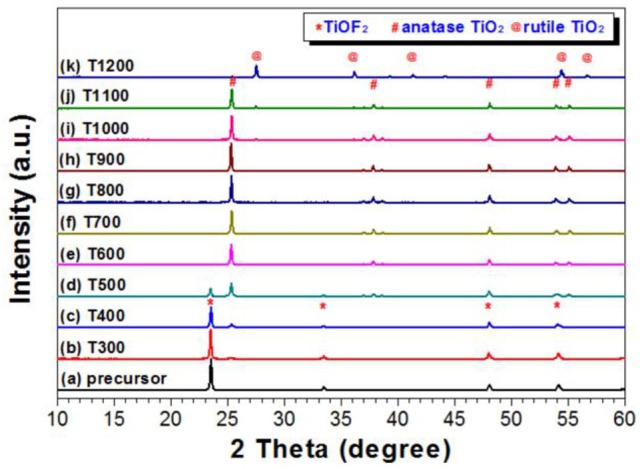
XRD patterns of the photocatalysts calcined at different temperatures.

Phase structure of the photocatalyst is important to the photoreactivity. Therefore, we used XRD to study the phase evolution of the photocatalyst during calcination. From Figure 1, a broad peak centering at 2θ = 23.4°, which corresponds to the (100) plane diffraction of TiOF_2_, was observed for the prepared precursor, and no any peak of TiO_2_ phases (A-TiO_2_ and R-TiO_2_) exists, indicating the successful synthesis of TiOF_2_(Huang et al., 2013). After calcination at 300°C for 2 h, the phase struccture of the sample (T300) almost keeps unchanged. With increase in the calcination temperature to 400°C, the peak intensity for TiOF_2_ decreases. Simultaneously, a small peak at 2θ = 25.3°, which corresponds to the (101) plane diffraction of A-TiO_2_, can be observed for T400 sample, indicating that phase transformation from TiOF_2_ to anatase begins. This phase transformation becomes obvious after calcination of TiOF_2_ at 500°C, and the prepared TiOF_2_ totally transforms into anatase TiO_2_ at 600°C.

It is proposed that the reaction for the heat-induced phase transformation from TiOF_2_ to TiO_2_ is as follows (Equation 1) (Zhao et al., 2018).

(1) $${2\text{TiOF}}_{2}\;+\text{ heat }\to \;{\text{TiO}}_{2}\;+\;{\text{TiF}}_{4}↑$$

Further increase in the calcination from 600 to 900°C, the peak intensity of A-TiO_2_ increases, indicating the enhanced crystallization. Meanwhile, the narrowing of the width for the (101) plane diffraction peak indicates the increase of A-TiO_2_ crystallite size. The peak intensity for the (101) peak of anatase TiO_2_ begins to decrease at calcination temperature of 1000°C, which is a sign of anatase-to-rutile phase transfermation. The formation of rutile phase is confirmed from the XRD pattern of the T1100 sample due to the formation of a small peak at 2θ = 27.3°, which corresponds to the (110) plane diffraction of R-TiO_2_. Only when the calcination temperature reaches 1200°C can all of the A-TiO_2_ transform into R-TiO_2_.

Usually, A-TiO_2_ nanocrystals begin transform into R-TiO_2_ at about 600°C. However, in the present study, the temperature for anatase-to-rutile transformation is as high as 1100°C, which indicates that these samples are promising to be used in hyperthermal situations.

### Evolution of the morphology

**Figure 2:**
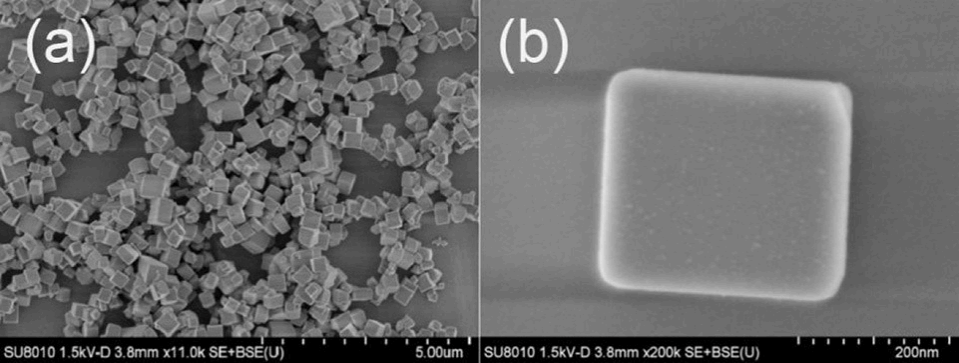
SEM images of the precursor TiOF_2_ at low **(a)** and high magnification **(b)**.

The SEM images of the precursor TiOF_2_ is shown in Figure 2. It can be clearly observed that these TiOF_2_ nanoparticles are in cubic shape and relatively monodispersed (Figure 2a), which is consistent with the reported literatures (Chen et al., 2012; Wang et al., 2012). From the high resolution SEM image shown in Figure 2b, we can estimate that the sidelength of the TiOF_2_ cube is about 250 nm.

**Figure 3:**
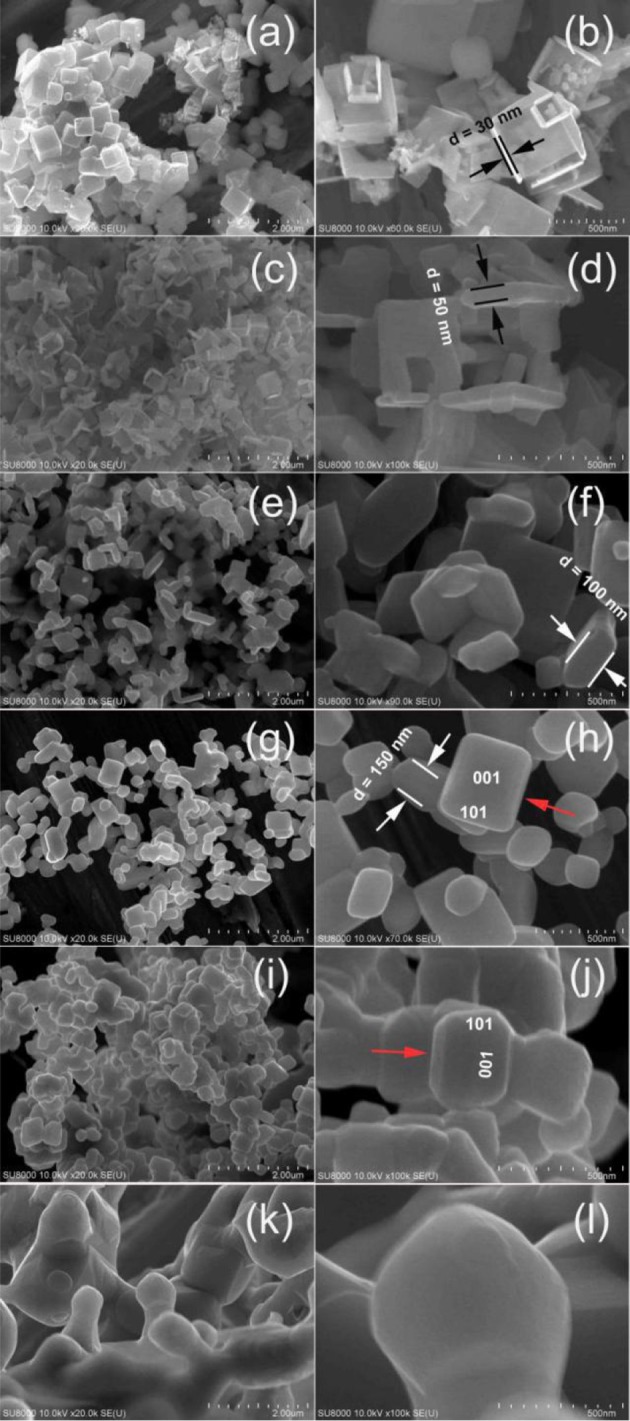
Comparison of the SEM images for T400 **(a,b)**, T500 **(c,d)**, T600 **(e,f)**, T700 **(g,h)**, T1100 **(i,j)**, and T1200 **(k,l)** samples.

After calcination of the precursor TiOF_2_ at 300°C, the morphology of the resulted sample (T300) almost keeps unchanged (SEM image not shown here). However, some nanoboxes with hollow interiors can be clearly seen from the SEM image of T400 sample (Figure 3a). These anatase TiO_2_ hollow nanoboxes (TiO_2_-HNBs) assemble from TiO_2_ nanosheets (TiO_2_-NSs) with exposed high energy (001) facets (Wen et al., 2011). The thickness of the TiO_2_-NSs is about 30 nm. It has been proven that high energy TiO_2_ nanosheets with exposed (001) facets can be prepared by using fluoride ions as shape-directing reagent (Yang et al., 2008; Lv et al., 2012). Then, it is not hard to understand the formation of high energy TiO_2_-NSs during calcination of TiOF_2_, a kind of fluorine-containing materials.

Since the sidelength of the obtained TiO_2_-HNBs is similar as these precursor TiOF_2_ cube, it is proposed that the formation of TiO_2_-HNBs assembly from TiO_2_-NSs is through a Ostwald Rippening process (Lou et al., 2008; Huang et al., 2013).

After calcination at 500°C, most of the TiOF_2_ cubes transform into TiO_2_-HNBs (Figure 3c). Figure 3d shows an enlarged SEM image of a broken TiO_2_ hollow nanobox, from which we can see that the thickness of the TiO_2_ nanosheet increases to about 50 nm, much thicker than that of T400 sample.

**Figure 4:**
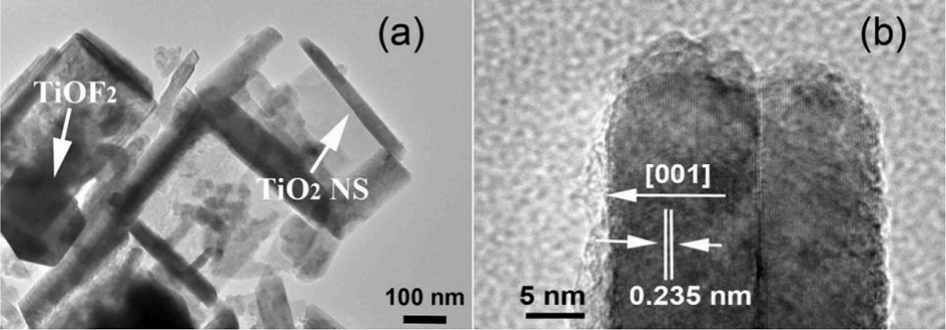
TEM image **(a)** and high-resolution TEM image **(b)** of T500 sample, arrows in **(a)** indicating the presence of TiOF_2_ cube and TiO_2_ nanosheet (TiO_2_-NS).

The structure of TiO_2_-HNBs was further confirmed by the corresponding TEM image of T500 sample (Figure 4a), from which we can observe the presence of some erected TiO_2_-NSs and a TiOF_2_ cube that has not totally transformed into anatase TiO_2_. This is consistent with the corresponding XRD characterization result (Figure 1d). From the side view HRTEM image of a discrete TiO_2_-NS (Figure 4b), we can clearly see the lattice spacing of 0.235 nm that parallels to the top and bottom facets, which corresponds to the (001) planes of A-TiO_2_ (Han et al., 2009). This confirms that the obtained TiO_2_-HNBs are assembly from high energy TiO_2_-NSs with exposed (001) facets (Wang et al., 2010).

When the calcination temperature increases to 600°C, we can see that almost all of the TiO_2_-HNBs decompose into discrete TiO_2_-NSs (Figure 3e). From the truncated biypramidal shape of a TiO_2_ nanocrystals (Figure 3f), we can estimate that the thickness of the TiO_2_-NSs is about 100 nm (T600 sample), which increases to ca. 150 nm for T700 sample (Figures 3g,h). Even calcined at 1100°C, some TiO_2_ nanosheets still keep bipyramidal shape (decahedron) with exposed (001) and (101) facets (Figures 3i,j), further indicating the thermal stability of high energy TiO_2_-NSs.

The bipyramidal shapes of TiO_2_ nanostructures disappear (Figures 3k,l) when calcination of TiOF_2_ cubes at 1200°C. This can be explained by the sintering of the sample due to phase transformation from A-TiO_2_ to R-TiO_2_ (Figure 1).

**Scheme 1:**
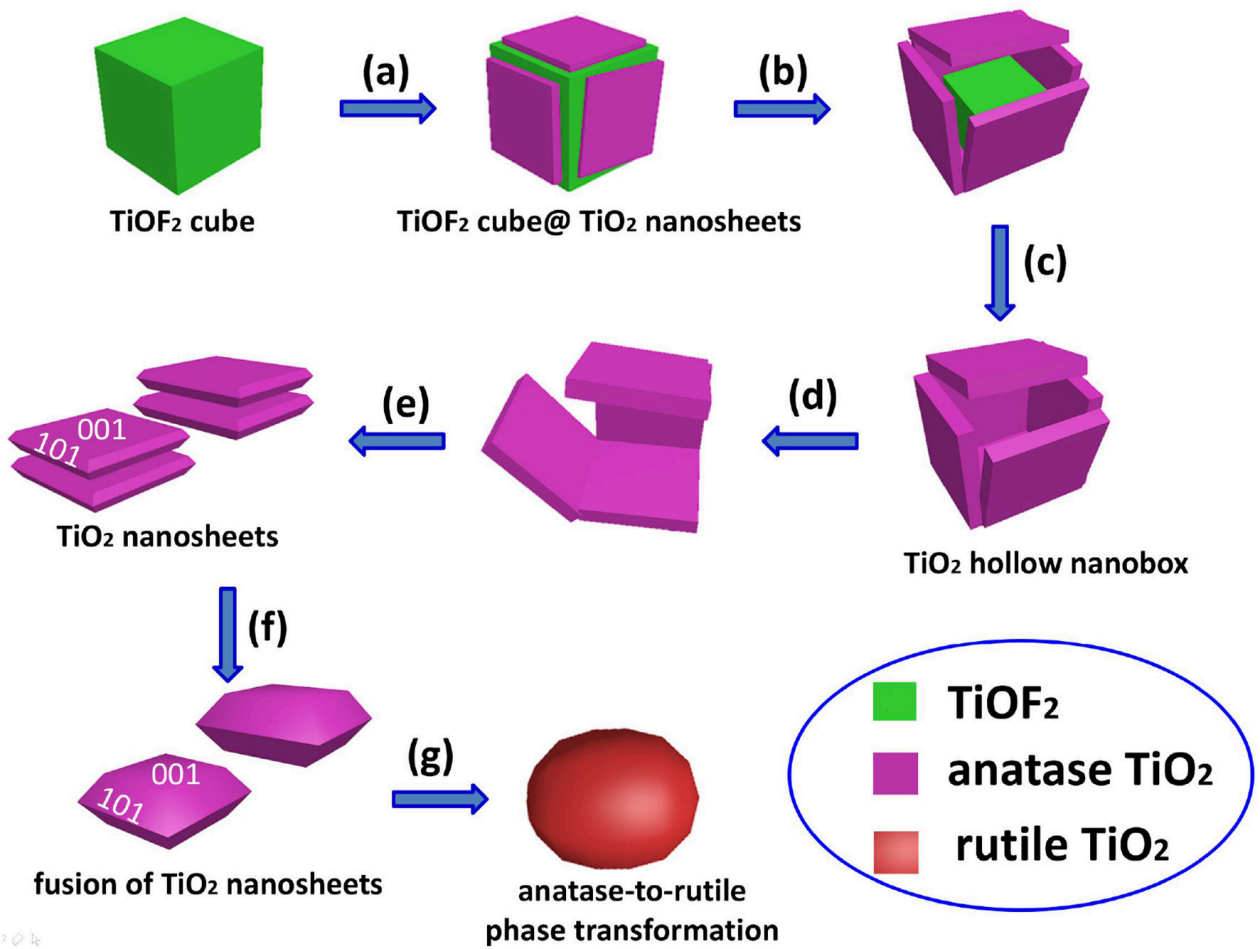
Morphology evolution and phase transformation of TiOF_2_ cube during calcination at different temperatures: **(a)** 300°C, **(b)** 400°C, **(c)** 500°C, **(d)** 600°C, **(e)** 700°C, **(f)** 1100°C, and **(g)** 1200°C.

By comparing the morphologies of TiO_2_-NSs from Figure 3, we can clearly see that heat treatment of TiOF_2_ cubes only results in the growth of TiO_2_ nanosheet along (001) direction. Scheme 1 illustrates the morphology evolution and phase transformation of TiOF_2_ cube during calcination.

### Uv-vis absorption and FTIR spectra

**Figure 5:**
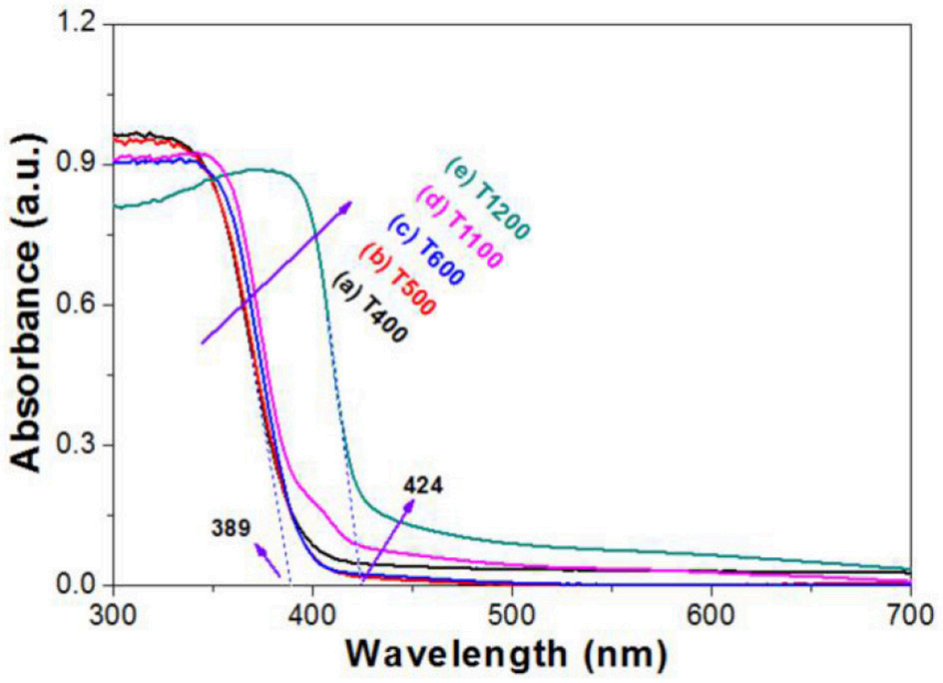
UV-vis absorption spectra of the photocatalysts.

Light-harvesting ability plays a very important role on the photoreactivity of the photocatalyst (Li et al., 2017b). Therefore, we compared the UV-vis absorption spectra of the samples. It can be seen that from Figure 5 that, when calcination temperature is below 1100°C, all samples possess similar absorption spectra (Figure 5). The onset of the UV-vis absorption spectrum for T400 sample is at 389 nm, corresponding to a bangap of 3.19 eV. However, the absorption edge was obviously red-shifted for T1200 sample. The onset of the spectrum for T1200 sample begins at 424 nm, corresponding to a bandgap of 2.92 eV, which can be ascribed to the phase transformation (Figure 1).

**Figure 6:**
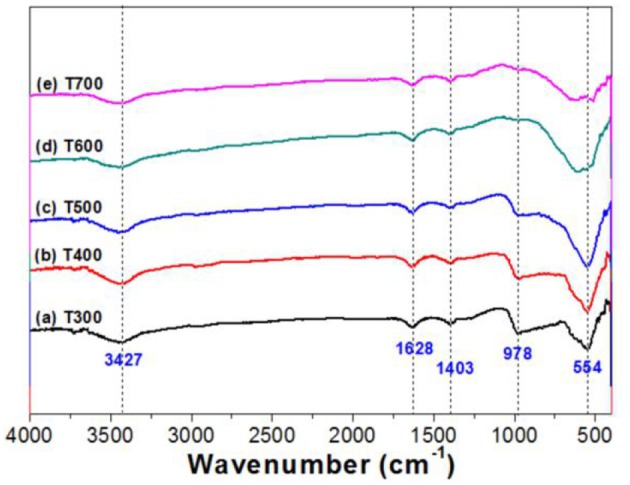
FTIR spectra of the photocatalysts.

Figure 6 compares the FTIR spectra of the photocatalysts treated at different calcination temperature. From which, it can be seen that all samples exhibit strong absorption peaks cenerting at about 3,427, 1,628, 1,403, and 554 cm^−1^. The peaks of 3,427 and 1,628 cm^−1^ originate from the -OH groups/H_2_O due to the adsorption of moisture from the air, while the peaks centering at about 1403 and 554 cm^−1^ originate from the vibration of Ti-O and O-Ti-O. Carefully view shows that there is a strong absorption peak at 978 cm^−1^, which decreases with increasing in the calcination temperature. This peak was attributed to the vibration of Ti-F bond of TiOF_2_. When calcination temperature increases to 600°C, the vibration of Ti-F disappears because of the complete phase transformation of TiOF_2_-to-anatase TiO_2_ (Figure 1; Zhao et al., 2018).

### Analysis of XPS spectrum

**Figure 7:**
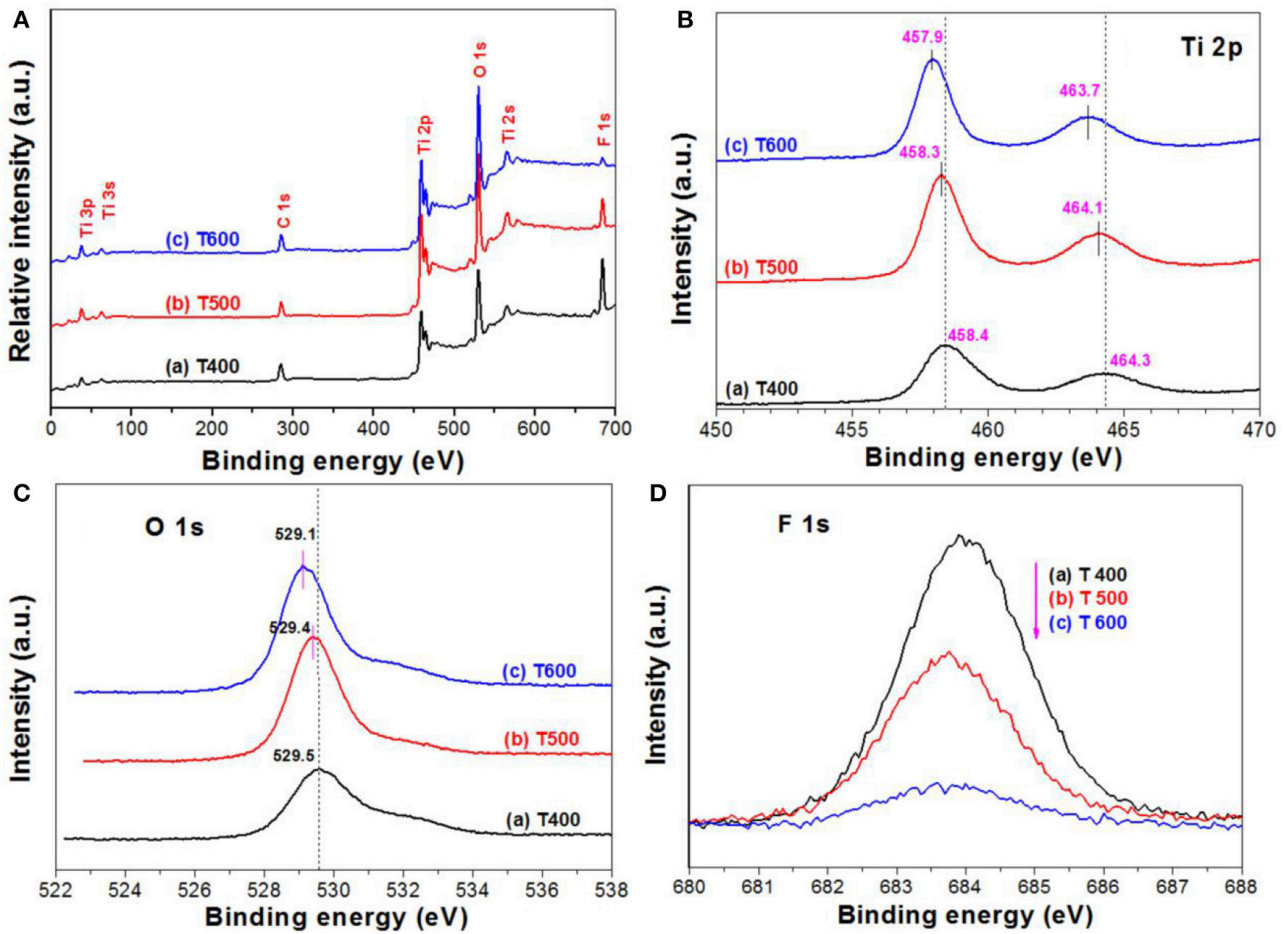
XPS survey spectra of the photocatalysts **(A)**, and the corresponding high resolution XPS spectra in Ti 2p **(B)**, O 1s **(C)**, and F 1s **(D)** regions, respectively.

Figure 7A compares the XPS survey spectra of photocatalysts for T400, T500, and T600 photocatalysts. From which, it can be observed that the XPS spectra are similar. All these samples contain titanium (Ti), oxygen (O), fluorine (F), and carbon (C) elements. The C element originates from the adventitious hydrocarbon from the XPS instrument itself. We can also be clearly see that the peak intensity of F element tends to decrease with increasing the calcination temperature from 400 to 600°C. The atomic ratios of F:Ti were determined to be 1.19 for T400 sample, 0.50 for T500 sample, and 0.17 for T600 sample, respectively. The steady decrease in F content with increase in the calcination is because of the TiOF_2_ to A-TiO_2_ phase transformation (Figure 1 and Equation 1), and the removal of adsorbed fluoride ions on the surface of A-TiO_2_.

Figures 7B,C show the high resolution XPS spectra in Ti 2p and O 1s regions, respectively. It can be seen that, both the binding energies for Ti 2p and O 1s of the samples steady decrease with increasing the calcination temperature. This is attributed to the TiOF_2_-to-TiO_2_ phase transformation (Figure 1 and Equation 1), and the formation of oxygen vacancy due to the removal of surface adsorbed fluoride ions over TiO_2_ upon calcination (Lv et al., 2011). It has been accepted that almost all of the fluoride ions adsorbed on the surface of high energy TiO_2_ nanosheets can be removed after calcination at 500°C.

The F 1s binding energy peak centering at about 684 eV (Figure 7D) is designated to the surface fluoride (Ti-F). No peak with binding energy of about 688.5 eV, corresponding to the lattice F^−^ of TiO_2_, is found in all the photocatalysts, indicating that calcination of TiOF_2_ cannot results in the doping of fluorine into anatase TiO_2_ (Yu et al., 2002). We can also clearly see that the peak intensity of F 1s steady decreases with increasing the calcination temperature, this can be attributed to the heat-induced phase transformation from TiOF_2_ to A-TiO_2_ and the desorption of surface adsorbed fluoride ions (Yang et al., 2008).

### Photocatalytic oxidation of acetone

Photocatalytic oxidation of acetone was used to monitor the photoreactivity of the prepared photocatalyst. The reaction for the photocatalytic oxidation of acetone is as follows (Equation 2).

(2) $${\left({\text{CH}}_{3}\right)}_{2}\text{CO }+\;{\text{4O}}_{2}\;=\;{\text{3CO}}_{2}\;+\;{\text{3H}}_{2}\text{O}$$

**Figure 8:**
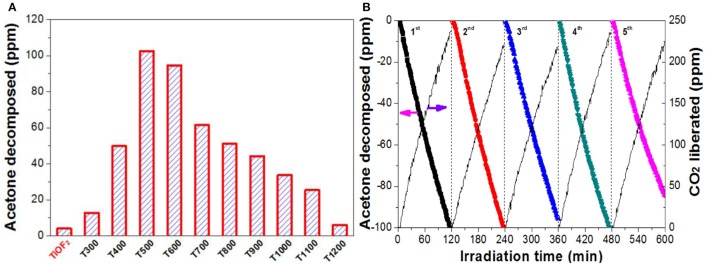
Comparison of the photoreactivity of the photocatalyst **(A)**, and the recycling use of T500 sample **(B)** in photocatalytic oxidation of acetone.

Figure 8A compares the relative photoreactivity of the photocatalyst by calculation the decomposed acetone within 2 h. It was found that the photocatalytic activity of the precursor TiOF_2_ can be neglected (only 4.0 ppm acetone was decomposed). The poor photocatalytic activity of TiOF_2_ is possibly due to its easy recombination of photo-generated carriers.

The photocatalytic activity of the photocatalyst increases with increase in the calcination temperature from 300°C (12.8 ppm) to 500°C (102.5 ppm), which is due to the production of A-TiO_2_. When further increase in the heat temperature from 600°C (94.6 ppm) to 1100°C (25.4 ppm), the photocatalytic activity of the photocatalyst steady decreases, possibly due to the decreased specific surface area (Table 1). Only 5.9 ppm of acetone was found to decompose when T1200 sample is used as photocatalyst. This is because of the complete A-TiO_2_ to R-TiO_2_ phase transformation (Figure 1) and smallest BET surface area (0.4 m^2^g^−1^).

Reusability of the photocatalyst is also important from the viewpoint of the practical applications (Liu et al., 2010). Therefore, we monitored the recycling use of T500 sample for 5 times in acetone oxidation (Figure 8B). It can be seen that no obvious reactivity decrease was observed for T500 sample even continual use for 5 times, indicating that T500 sample is potential to be used in practical applications.

### Mechanism

**Figure 9:**
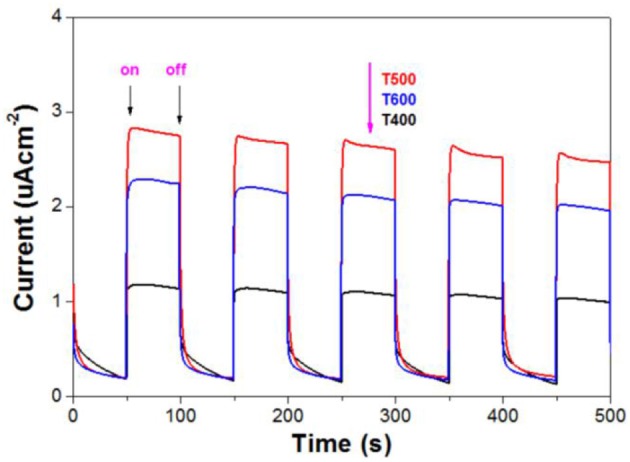
Photocurrents of the photocatalysts.

The value of photocurrent is usually used to evaluate the ability to generate and transfer of charge carriers for illuminated semiconductor photocatalyst (Cheng et al., 2018; Huang et al., 2018). The photocurrent of the prepared photocatalyst was therefore tested for several on-off cycles (Figure 9). It can be seen that, when irradiation of the TiO_2_ film electrodes, obvious prompt photocurrent signals are produced, which exhibit good reproducibility. When the lamp is turned off, the photocurrent value for all the TiO_2_ film electrodes are instantaneously close to zero. We can also see, with increasing the calcination temperature from 400 to 600°C, the photocurrent of the photocatalyst increases first and then decreases. The photocurrent of T500 sample is the as high as 2.8 uAcm^−2^, which is much higher than that of T400 (1.1 uAcm^−2^) and T600 (2.3 uAcm^−2^) samples. The photoreactivity of semiconductor photocatalyst is closely related to the efficiency of the separation of the photo-generated electron-hole pairs (Fu et al., 2008). So it is safe to predict that the photoreactivity of T500 is higher than that of T400 and T600 samples, which keeps in line with the experimental results (Figure 8).

It has been accepted that fluoride ions show strong affinity to titanium (Equation 3), and the photoreactivity of TiO_2_ nanocrystals can be obviously improved after the introduction of surface fluorination (Minero et al., 2000; Xu et al., 2007; Cheng et al., 2008). According to the study of Yu et al., the difference in surface energy makes the photo-generated electrons and holes migrate to (101) and (001) facets, respectively (Yu et al., 2014). The CB electrons aggregated in (101) facets are captured by surface adsorbed oxygen to produce super oxygen radicals (${\text{O}}_{2}^{\cdot -}$), while the VB holes aggregated on the (001)facets are transfer into hydroxyl radicals (·OH). Both ${\text{O}}_{2}^{\cdot -}$ and ·OH are important reactive oxygen species (ROSs) for the degradation of organics. When compared with pristine TiO_2_, surface fluorination of TiO_2_ changes the state of the formed hydroxyl radicals from surface bounded ·OH radicals (Equation 6) to mobile ·OH radicals (·OH_free_). This is because the displacement of fluoride to surface OH^−^ groups (Equation 3) induces the direct oxidization solvent water by holes (Equation 7).

(3) $$\equiv \text{Ti}-\text{OH }+\;{\text{F}}^{-}\;\to \;\equiv \text{Ti}-{\text{F+OH}}^{-}$$

(4) $${\text{TiO}}_{2}\;+\text{ hv }\to \;{\text{TiO}}_{2}\;({\text{h}}^{+}\cdot \cdot \cdot {\text{e}}^{-})$$

(5) $${\text{O}}_{2}\;+\;{\text{e}}^{-}\;\to \;{{\text{O}}_{2}}^{\cdot -}$$

(6) $$\equiv \text{Ti}-\text{OH }+\;{\text{h}}^{+}\;\to \;\equiv \text{Ti}\cdot \cdot \cdot \text{OH}$$

(7) $$\equiv \text{Ti}-\text{F }+\;{\text{H}}_{2}\text{O }+{\text{h}}^{+}\;\to \;\equiv \text{Ti}-\text{F }+\;\cdot {\text{OH}}_{\text{free}}$$

**Scheme 2:**
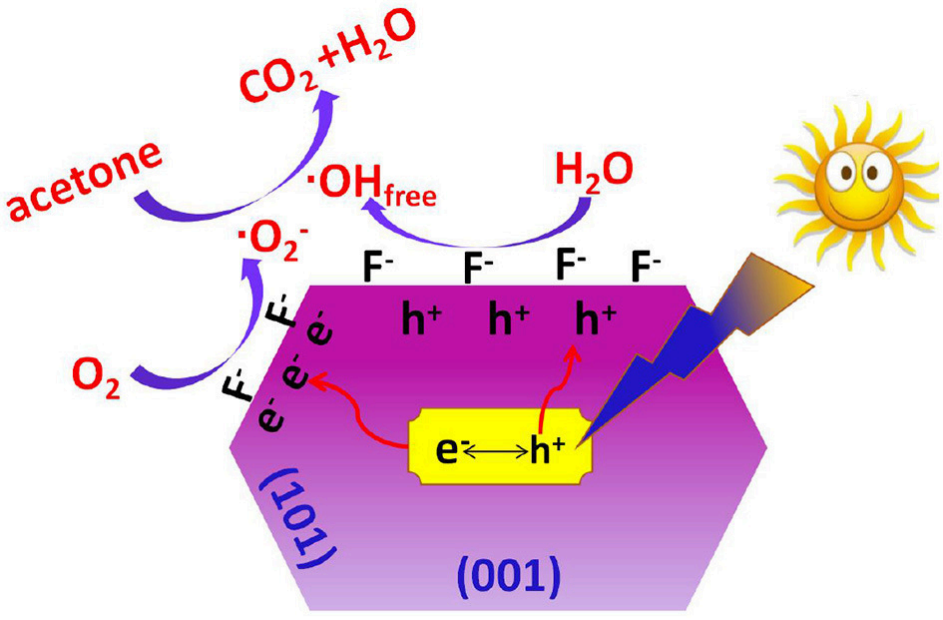
Proposed mechanism for the enhanced photocatalytic activity of surface fluorinated TiO2 nanosheets with exposed high energy (001) facets toward acetone oxidation.

As free ·OH radical are more active than surface bounded ·OH radicals, the oxidation of acetone into CO_2_ and H_2_O is greatly enhanced due to the attacks of super oxygen radicals and free ·OH radicals (Equation 8). The proposed mechanism for the enhanced photoreactivity of surface fluorinated TiO_2_ nanosheet toward acetone oxidation is shown in Scheme 2.

(8) $${\text{C}}_{3}{\text{H}}_{6}\text{O }+\;{{\text{O}}_{2}}^{\cdot -}/\cdot {\text{OH}}_{\text{free}}\;\to \;{\text{CO}}_{2}\;+\;{\text{H}}_{2}\text{O}$$

Recently, significantly growing interest has been devoted to studying of hierarchical nanostructures due to their unique properties and widespread potential applications (Lou et al., 2008). For example, when compared with solid spheres, TiO_2_ hollow spheres usually shows much higher photocatalytic activity mainly because they possess better light-harvesting ability (Li et al., 2009). In the present study, the hierarchically structured T500 sample, that is TiO_2_ hollow nanobox assembly from TiO_2_ nanosheets should also benefit the use of light (Figure 5), enhancing the photoreactivity.

However, the photoreactivity of TiO_2_-NSs steady decrease with increase in the calcination temperature from 500 to 1100°C due to the collapse of the hierarchical TiO_2_ hollow nanobox and the removal of surface adsorbed fluorine, leaving surface oxygen vacancy (Figure 8; Cheng et al., 2018).

(9) ≡Ti−F + heat → ≡Ti−□

**Figure 10:**
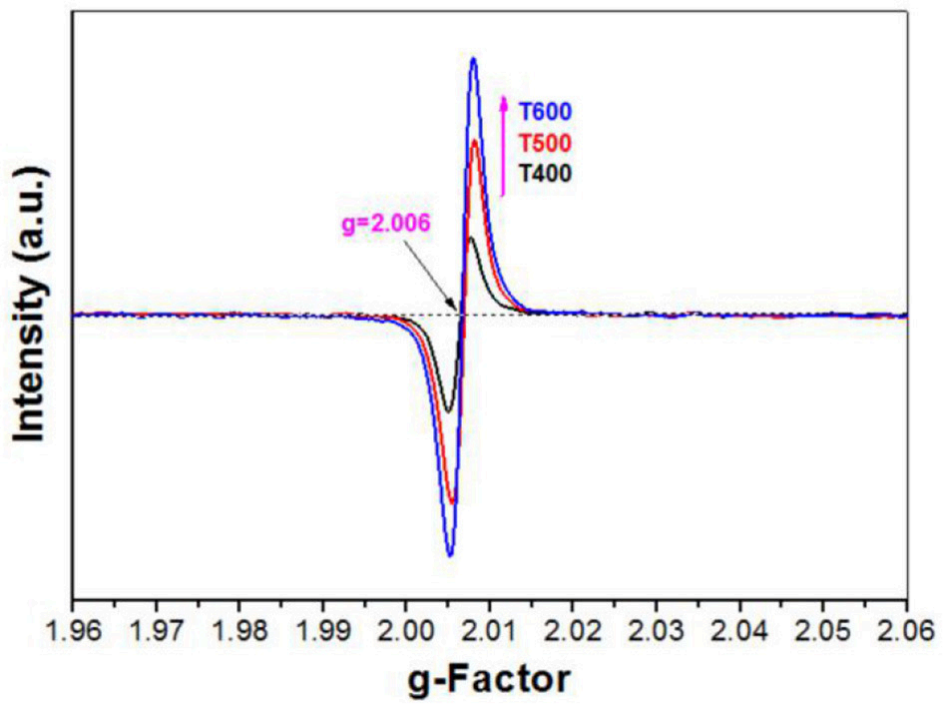
EPR spectra of the photocatalysts.

The formation of surface oxygen vacancy was proved by electron paramagnetic resonance (EPR) technique. It was found that the signal intensity for oxygen vacancy of the photocatalyst increases with increase in the calcination temperature from 400 to 600°C (Figure 10) due to the heat-induced desorption of surface adsorbed fluoride ions (Equation 9).

(10) $$\equiv \text{Ti}-\text{OH }+\text{ HO}-\text{Ti}\equiv \to \equiv \text{Ti}-\text{O}-\text{Ti}\equiv \;+\;{\text{H}}_{2}\text{O}$$

(11) ≡Ti−□ + □−Ti≡ → no reaction

It was believed that only when the crystalline size of the nanocrystal is larger than a critical size can phase transformation begin (Padmanabhan et al., 2007; Periyat et al., 2008, 2009). Therefore, the growth of TiO_2_ nanocrystal is a prerequisite before the phase transformation of A-TiO_2_ to R-TiO_2_. The growth of pristine TiO_2_ nanocrystal is relatively easy by formation of =Ti-O-Ti= chain between two neighboring TiO_2_ nanoparticles (Equation 10). However, the growth of the =Ti-O-Ti= chain is prevented due to the formation of surface oxygen vacancy (Equation 11). Only when the lattice oxygen is diffused from the bulk to the surface of TiO_2_ nanosheet with oxygen vacancy at high temperaure can the fusion of neighboring TiO_2_-NSs become possible (Lv et al., 2011). Therefore, it is not hard to understand the super thermal stability of TiO_2_-NSs prepared by calcination of TiOF_2_ cubes.

After the removal of surface adsorbed fluoride ions by calcination, these TiO_2_-NSs prefer to aggregate and grow along (001) direction to reduce the high surface energy (Lv et al., 2011). Then we can clearly observe the steady increase in the thickness of TiO_2_-NSs (Figure 3).

## Conclusions

TiO_2_ nanosheets with high thermal stability were prepared by calcination of TiOF_2_ cubes. The anatase-to-rutile phase transformation temperature reaches as high as 1100°C. 500°C-calcined sample shows the highest photoreactivity toward acetone oxidation due to the surface fluorination. The high thermal stability of TiO_2_ nanosheets is ascribed to the introduction of surface oxygen vacancy after removal of the surface adsorbed fluoride ions, which prevents the growth of TiO_2_ nanosheets. The present study provide a novel way in design of thermally stable materials.

## Author contributions

TS and YD performed the experiments. KL planned the project, designed the experiments, and wrote the manuscript. ZH, QL and XL assisted in the analysis and interpretation of the data. ML revised the manuscript.

### Conflict of interest statement

The authors declare that the research was conducted in the absence of any commercial or financial relationships that could be construed as a potential conflict of interest.
